# Mixed-Supervised Learning for Cell Classification

**DOI:** 10.3390/s25041207

**Published:** 2025-02-16

**Authors:** Hao Sun, Danqi Guo, Zhao Chen

**Affiliations:** 1 School of Computer Science and Technology, Donghua University, Shanghai 201620, China; 2232835@mail.dhu.edu.cn (H.S.); 2212622@mail.dhu.edu.cn (D.G.); 2 Department of Computer Science, University of Warwick, Coventry CV4 7AL, UK

**Keywords:** cell classification, mixed-supervised learning, human-in-the-loop

## Abstract

Cell classification based on histopathology images is crucial for tumor recognition and cancer diagnosis. Using deep learning, classification accuracy is hugely improved. Semi-supervised learning is an advanced deep learning approach that uses both labeled and unlabeled data. However, complex datasets that comprise diverse patterns may drive models towards learning harmful features. Therefore, it is useful to involve human guidance during training. Hence, we propose a mixed-supervised method incorporating semi-supervision and “human-in-the-loop” for cell classification. We design a sample selection mechanism that assigns highly confident unlabeled samples to automatic semi-supervised optimization and unreliable ones for online annotation correction. We use prior human annotations to pretrain the backbone and trustworthy pseudo labels and online human annotations to fine-tune the model for accurate cell classification. Experimental results show that the mixed-supervised model reaches overall accuracies as high as 86.56%, 99.33% and 74.12% on LUSC, BloodCell, and PanNuke datasets, respectively.

## 1. Introduction

Cell classification plays an important role in areas such as pathology diagnosis, cancer research, and drug development. Precise identification and classification of cells are crucial for comprehending cellular behavior, diagnosing diseases, and developing effective treatments. Traditionally, cell classification has relied heavily on manual observation and expert interpretation, which are not only time-consuming but also susceptible to subjective biases. With the rapid advancement of computer vision technology, numerous methods for automatic analysis of pathology images have been applied.

Deep learning methods can be broadly categorized into three main types based on their level of supervision: unsupervised [1,2], fully supervised [3,4,5,6,7], and semi-supervised [8,9,10] approaches. Unsupervised methods are data-driven, yielding results that may not be immediately understandable to human users. Most deep learning methods use large amounts of manually annotated data for training in a fully supervised manner, which is labor-intensive and limits the model’s adaptability to new data. As hospitals and pathological diagnostic institutions produce a large number of microscopic pathological slides every day without accurate manual annotations, semi-supervised online learning methods have emerged. Compared to fully supervised models, semi-supervised models simultaneously use labeled data and unlabeled data for training, adapting to the diversity of open data and making predictions on new data during training. Semi-supervised methods mainly consist of three types [11]: consistency regularization-based methods [12], generative model-based methods [13,14], and pseudo-labeling-based methods [15,16]. For instance, ref. [12] introduced distance correlation to minimize the correlation between feature representations of different views of the same image.

However, semi-supervised methods often encounter challenges with complex datasets, as they may learn irrelevant or harmful features. Meanwhile, pathologists not only expect to quickly review slides through algorithms but also need to correct algorithm errors promptly [17]. To address these issues and align with practical needs, the “human-in-the-loop” approach has been proposed. This method focuses on selecting data based on specific criteria to optimize the model’s predictive performance [18]. Various methods have been developed to quantify the informativeness of samples relative to a given model and its underlying distribution. These methods can generally be grouped into four categories: uncertainty, representativeness, generative adversarial networks (GANs) for informativeness, and learning active learning. The order reflects the level of human interpretability each method offers, ranging from the most interpretable to the least. In Table 1, we present several classical algorithms for these methods.

The introduction of the “human-in-the-loop” approach allows experts to intervene during training by annotating difficult samples, thereby improving model accuracy with minimal additional effort [18]. For instance, ref. [19] used concept-based interpretability methods like concept activation vectors (CAV) and concept activation regions (CAR) to identify weaknesses in classifier training. Clinicians can then use these insights to determine which concepts or categories need improvement and adjust the dataset to optimize classifier performance. Similarly, ref. [20] involved pathologists throughout the entire model prediction process. This included reviewing and correcting automated segmentation results, guiding the classification process, and providing feedback on interpretability. The pathologist’s expertise not only helped refine the model’s output but also contributed to hyperparameter optimization. Unlike explicit sample selection and processing, ref. [21] proposed a new deep learning method, TOP-GAN, which combines transfer learning and GANs to accurately classify healthy and cancer cell lines using limited labeled data by generating additional training images from sperm cells.

**Table 1:** Human-in-the-loop for cell classification.

Evaluating Informativeness	Principle	Examples
Uncertainty	Predictions with higher uncertainty offer greater potential for improving the model when their ground truth is incorporated into the training set.	[22,23]
Representativeness	Encourage selection strategies to sample from different areas of the distribution, and to increase the diversity of samples	[19,24]
Generative adversarial networks for informativeness	GANs are used for image synthesis and data augmentation, enhancing active learning by expanding limited datasets	[21,25]
Learning active learning	Methods that learn which samples are most informative based on previous selection outcomes.	[20,26]

The methods mentioned above have shown effectiveness in the field of medical image processing. However, they still fall short in terms of human interpretability. In contrast, interpreting model prediction probabilities as confidence provides a straightforward and highly interpretable means of measuring sample uncertainty. Additionally, confidence not only effectively selects complex samples to enhance the model’s reasoning ability but also reduces the computational cost for processing credible samples, thereby accelerating the training process.

Therefore, we propose a mixed-supervised tumor cell classification framework (MIX-CC) based on a hard sample selection mechanism. It introduces “human-in-the-loop” for online annotation to semi-supervised cell classification. Our contributions are as follows. (1) The proposed method classifies cells in unlabeled images by a sample selection mechanism that allows for online correction of unreliable predictions and automatic optimization using reliable pseudo-labels of the originally unlabeled images. This mechanism is particularly well-suited for large-scale, open pathology datasets, allowing for broader real-world applications. (2) It improves classification accuracy by pretraining the backbone by prior human annotations and refining the model using reliable pseudo labels and online human annotations. This process facilitates precise interpretation of open data, making it ideal for large-scale, practical applications.

Compared to baseline algorithms, our proposed MIX-CC achieves the best performance across three different datasets, showing significant improvements over both fully supervised and semi-supervised learning methods. The remainder of this paper is organized as follows. In Section 2, we provide details of various components in the proposed framework. In Section 3, we present results demonstrating the effectiveness of the proposed framework. Section 4 presents the discussion, while Section 5 concludes the study.

## 2. Materials and Methods

This section provides a detailed description of the dataset, the proposed framework, and programming environment in which the experiment was conducted.

### 2.1. DataSet

**Figure 1:**
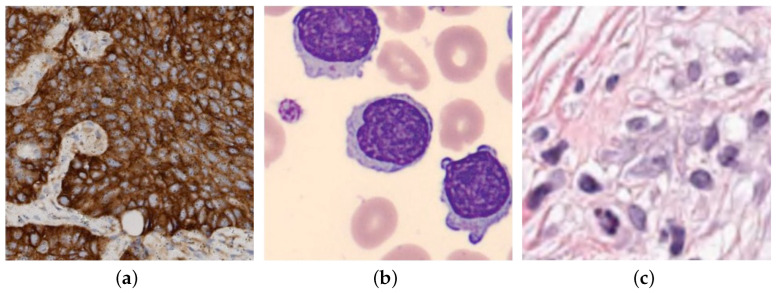
Samples from (**a**) LUSC, (**b**) BloodCell, and (**c**) PanNuke Datasets.

The performance of MIX-CC is evaluated across three pathology image datasets: LUSC, BloodCell, and PanNuke, as shown in Figure 1. The datasets are partitioned into labeled sets, validation sets, and unlabeled sets. The labeled set simulated historical data, while the unlabeled set simulated open data, with the validation set employed for model tuning. The specifics of each dataset are detailed below:

(1) Lung Squamous Cell Carcinoma Dataset (LUSC) [27]: LUSC is a private dataset, provided by Histo Pathology Diagnostic Center, Shanghai, China (HISTO). It contains 44 immunostained PD-L1 images with 1000 × 1000 pixels obtained from 4 whole slide images scanned by KF-PRO-120 (0.2481 μm/pixel, 40× objective magnification), as shown in Figure 1a. Multiple experts manually labeled the cell nuclei, including two types of tumor cells: positive cells (Positive) and negative cells (Negative). LUSC is randomly divided in a ratio of 7:1:2, comprising 35 labeled images and 9 unlabeled images. Cell image patches in LUSC are set to dimensions of 76 × 76.

(2) BloodCell Dataset [28]: Originating from the Ruijin Hospital affiliated with Shanghai Jiao Tong University School of Medicine, it consists of 1326 color images of blood smears, each with dimensions of 360 × 360 pixels, as shown in Figure 1b. These include three types of cells: blast cells (Blast), lymphocytes (Lymphocyte), and lymphoma cells (Lymphoma). BloodCell is randomly split at a ratio of 6:1:3, with 795 images in the labeled set and 399 images in the unlabeled set. The cell image patches in the BloodCell dataset are set to dimensions of 200 × 200.

(3) Pan-Cancer Histology Dataset (PanNuke) [29,30]: PanNuke is published by the University of Warwick. It is a complex, multi-class cell dataset containing 7501 H&E stained images with 256 × 256 pixels from 19 different tissue types, as shown in Figure 1c. Each image has pixel level labels for cell segmentation and classification. These include five types of cells: neoplastic cells (Neoplastic), inflammatory cells (Inflammatory), connective tissue cells (Connective Tissue), dead cells (Dead), and non-neoplastic cells (Non-neoplastic). It is divided in a ratio of 5:1:4, comprising 3950 labeled images and 3161 unlabeled images. The size of cell image patches in PanNuke are set to 60 × 60.

### 2.2. MIX-CC Framework

**Figure 2:**
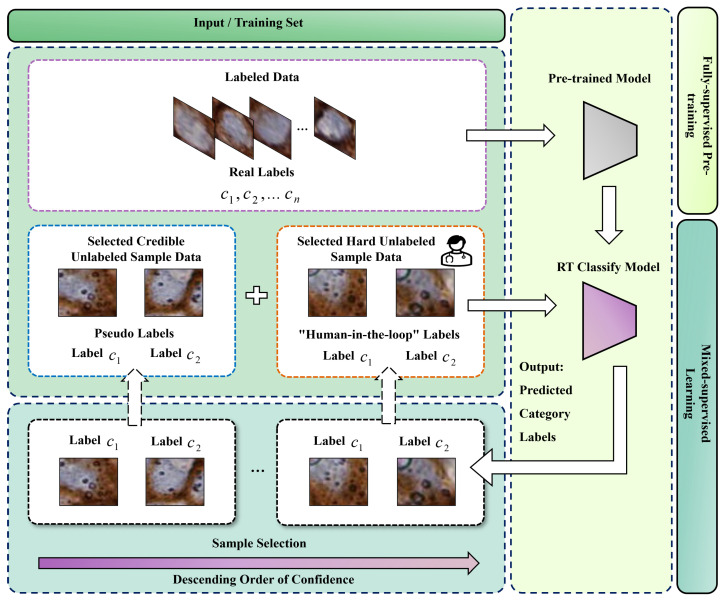
Mixed-supervised tumor cell classification framework based on difficult sample screening mechanism (MIX-CC).

The proposed MIX-CC framework, depicted in Figure 2, is designed with two stages: the fully supervised pretraining stage and the mixed-supervised online learning stage. During the fully supervised pretraining phase, a classification network is trained with labeled data, establishing a strong preliminary feature representation. In the subsequent mixed-supervised learning stage, the “human-in-the-loop” mechanism is employed to dynamically annotate challenging and ambiguous samples from the unlabeled set. Coupled with the credible sample selection and iterative training set updating mechanisms, MIX-CC continuously optimizes the model, enhancing its accuracy and robustness while making refined category predictions for cells in new and diverse images. This approach ensures high adaptability and precision in cell classification tasks.

Let ${\underline{X}}_{i}\in R^{H\times W\times B}$ denote the input cell image patch obtained from the data preprocessing stage, where $i=1,2,\ldots n^{\left(L\right)}$, $n^{\left(L\right)}$ denotes the number of images in the training set, *H*, *W*, and *B* represents width, height, and number of channels, respectively, B=3. As the iterations of the mixed-supervised mechanism progress, $n^{\left(L\right)}$ will gradually increase. The dataset is divided into a labeled set ${\left\{{\underline{X}}_{j}^{\left(L\right)},y_{j}\right\}}_{j=1}^{n_{0}^{\left(L\right)}}$ and an unlabeled set ${\left\{{\underline{X}}_{j}^{\left(U\right)}\right\}}_{j=1}^{n_{0}^{\left(U\right)}}$, where ${\underline{X}}_{j}^{\left(L\right)}$ represents the labeled image, ${\underline{X}}_{j}^{\left(U\right)}$ represents the unlabeled image, $y_{j}$ represents the ground truth of labeled image, $n_{0}^{\left(L\right)}$, and $n_{0}^{\left(U\right)}$ denotes the initial number of labeled and unlabeled samples. In the hard sample selection mechanism, we define the number of unlabeled samples randomly selected in each semi-supervised iteration as $N_{i}$, the number of “credible” unlabeled samples selected in each as $K^{\left(\mathrm{Conf}\right)}$, the number of “hard” unlabeled samples selected in each as $K^{\left(\mathrm{Hard}\right)}$, and the remaining unlabeled samples as $K^{\left(\mathrm{Rest}\right)}$. Finally, output the prediction obtained from online classification of the selected unlabeled sample $m^{\left(U\right)}$ as result ${\left\{{\underline{X}}_{j}^{\left(U\right)},{\hat{y}}_{j}^{\left(U\right)}\right\}}_{j=1}^{m^{\left(U\right)}}$ and the classes of the remaining unlabeled samples obtained from offline classification as result ${\left\{{\underline{X}}_{j}^{\left(U\right)},{\hat{y}}_{j}^{\left(U\right)}\right\}}_{j=1}^{K^{\left(\mathrm{Rest}\right)}}$. In this context, ${\left\{{\underline{X}}_{j}^{\left(U\right)},{\hat{y}}_{j}^{\left(U\right)}\right\}}_{j=1}^{m^{\left(U\right)}}$ serves as choosing samples and their pseudo-labels are incorporated into the current training set through an update mechanism, contributing to model optimization in the next iteration cycle. The symbols and notations used in this description are summarized in Table 2 for better clarity and reference.

### 2.3. Backbone

Selecting an appropriate backbone is crucial for improving cell classification performance, as its design and pretraining are essential for the model’s generalization on complex datasets. The proposed MIX-CC framework exhibits a certain degree of flexibility, allowing the utilization of any classification model as the backbone. In this paper, ResNet [31] is employed as the backbone. It is utilized for both the fully supervised pretraining stage and the mixed-supervised training stage. To fully exploit the potential of MIX-CC, different ResNet variants pretrained on the ImageNet dataset [32], including ResNet18 [31] and ResNet50 [31], are employed for different datasets based on empirical observations. Specifically, ResNet18 yields better performance on certain datasets, while ResNet50 performs more effectively on others. As illustrated by Figure 2, the backbone for both fully supervised pretraining and mixed-supervised training stages of MIX-CC remains consistent.

### 2.4. Fully Supervised Pretraining

**Figure 3:**
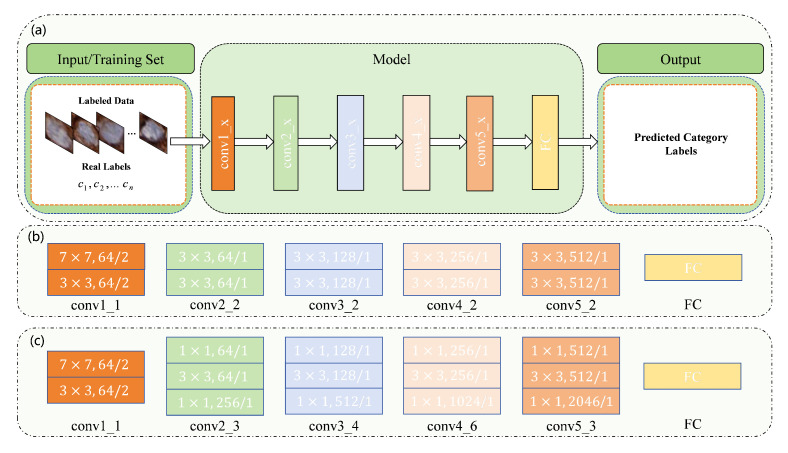
Conceptual diagram of the fully supervised pretraining phase. (**a**) is the framework diagram of the fully supervised pretraining phase, while (**b**) and (**c**) are the structure diagrams of the ResNet18 and ResNet50 backbones that we use, respectively.

To enhance the reliability of pseudo-labels and improve the efficiency and effectiveness of mixed-supervised training, MIX-CC first utilizes the labeled set ${\left\{{\underline{X}}_{j}^{\left(L\right)},y_{j}\right\}}_{j=1}^{n_{0}^{\left(L\right)}}$ for initialization, feeding it into the ResNet18 or ResNet50 backbones to produce pretrained classification networks, as shown in Figure 3. At this stage, we use the ground truth and model predictions to compute the cross-entropy loss for model optimization, as shown in Equation (1).

(1) $$\ell^{\left(\mathrm{fully}\right)}=-\frac{1}{n_{0}^{\left(L\right)}}\sum_{j=1}^{n_{0}^{\left(L\right)}}y_{j}\log\left({\hat{y}}_{j}\right)$$

These pretrained models serve as a high-quality foundation for generating pseudo-labels in the subsequent mixed-supervised phase.

### 2.5. Mixed-Supervised Online Learning

**Figure 4:**
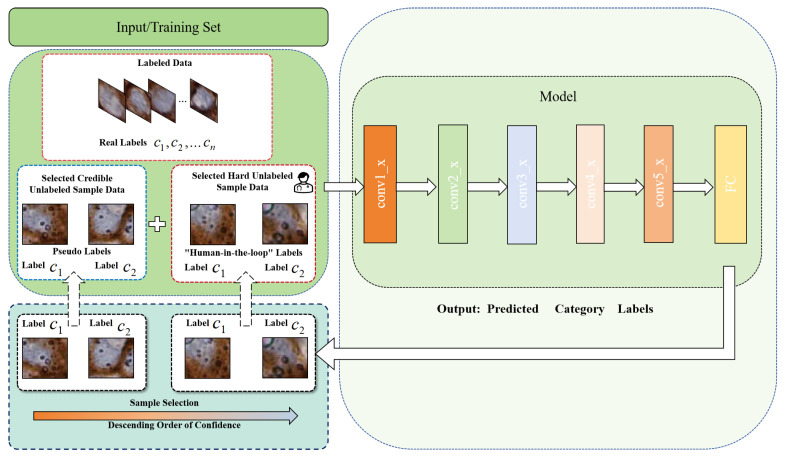
The framework diagram of the mixed-supervised online learning phase.

After the fully supervised pretraining stage, the classification networks gain initial feature representation capabilities. However, the presence of challenging samples in the open data can interfere with the model, limiting its generalization. Therefore, the framework initiates mixed-supervised training after fully supervised pretraining, as shown in Figure 4. The mixed-supervised online learning stage comprises three mechanisms: sample selection mechanism, dynamic training set updating mechanism and model iterative training mechanism. The framework takes the labeled set ${\left\{{\underline{X}}_{j}^{\left(L\right)},y_{j}\right\}}_{j=1}^{n_{0}^{\left(L\right)}}$ and unlabeled set ${\left\{{\underline{X}}_{j}^{\left(U\right)}\right\}}_{j=1}^{n_{0}^{\left(U\right)}}$ as inputs, assuming that pathologists annotate “hard” samples identified by the model in each iteration. The model automatically identifies “credible” and “hard” samples by evaluating the uncertainty in its predictions, which is derived from prediction confidence. “Credible” samples are those with higher confidence, while “hard” samples are typically those that the model finds difficult to classify, often due to factors such as unseen variations, ambiguous boundaries, or underrepresented categories in the training data. These samples tend to have low confidence scores and may represent rare or complex cases that challenge the model’s existing knowledge. By incorporating pseudo-labeled samples and hard samples annotated by pathologists into the training set, the model iteratively refines its ability to handle difficult samples and enhance feature representation. This iterative process not only improves the model’s performance on hard-to-classify cases but also facilitates the simultaneous prediction of categories for unlabeled samples during training. Consequently, the framework minimizes manual annotation costs while ensuring annotation accuracy.

In the mixed-supervised stage, we use the cross-entropy loss to optimize the function, just as in the pretraining stage, as shown in Equation (2). It is important to note that, at this stage, $y_{j}$ represents not only the labels of the labeled data but also includes pseudo-labels for “credible” samples and online annotations for “hard” samples, both selected from the unlabeled data through the sample selection mechanism.

(2) $$\ell^{\left(\mathrm{mixed}\right)}=-\frac{1}{n^{\left(L\right)}}\sum_{j=1}^{n^{\left(L\right)}}y_{j}\log\left({\hat{y}}_{j}\right)$$

After each iteration of training, MIX-CC conducts online testing on the unlabeled samples selected to update the training set and records the results as the classification results for this portion of the samples. Such a human–computer interaction mechanism enhances the model’s predictive capabilities.

### 2.6. Algorithm Description

The MIX-CC framework, detailed in Algorithm 1, consists of two stages: fully supervised pretraining and mixed-supervised online learning. During the pretraining and mixed-supervised stages, the model is optimized using gradient descent to update its parameters. The sample selection and online labeling mechanism used in the mixed-supervised learning stage are described in Algorithm 2.
**Algorithm 1:** Mixed-supervised cell classification.
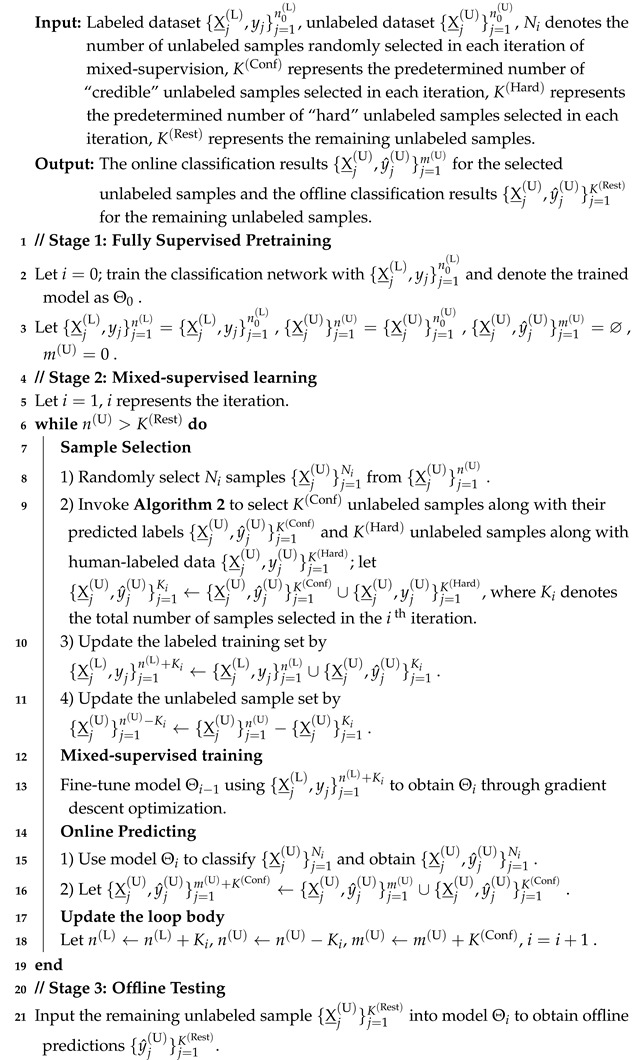

**Algorithm 2:** Sample Selection and Online Labeling.
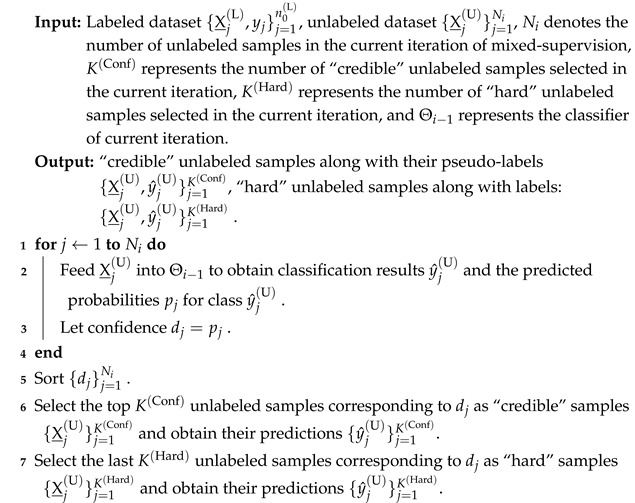


### 2.7. Setup

#### 2.7.1. Competing Algorithm Settings

This study employs simulated expert annotations to mimic pathology expert online annotating, comparing the performance of ResNet18, SimCLR [33] and ResNet50 in fully supervised for classification tasks on different datasets. For both BloodCell and LUSC, UNeXt [34] is utilized for cell segmentation map prediction, employing ResNet18 for both fully supervised and semi-supervised learning. Conversely, for the PanNuke dataset, HoVer-Net [3] is employed for cell segmentation map prediction, utilizing ResNet50 for both fully supervised and semi-supervised learning stages. Table 3 provides a summary of the competing algorithm settings.

#### 2.7.2. Hyperparameter Setting

The experiments mainly consist of hyperparameters for both the fully supervised pre-training stage and the mixed-supervised learning stage. Different datasets require adjustments in hyperparameters. The details of the semi-supervised stage are as follows.

For LUSC, the initial learning rate for the semi-supervised stage is 0.0003. Every 5 training epochs, the learning rate is reduced to 0.6 of its current value. Batch size is 64, $N_{i}$ is 300. $K^{\left(\mathrm{Conf}\right)}$, $K^{\left(\mathrm{Hard}\right)}$ and $K^{\left(\mathrm{Rest}\right)}$ is 20, 20 and 0, respectively.

For BloodCell, the initial learning rate for the semi-supervised stage is 0.0002. Every 5 training epochs, the learning rate is decreased to 0.8 of its current value. Batch size is set to 64. The number of candidate unlabeled samples randomly selected per iteration $N_{i}$ is 200. The preset number of credible samples per iteration $K^{\left(\mathrm{Conf}\right)}$, the number of hard samples annotated online per iteration $K^{\left(\mathrm{Hard}\right)}$ and the remaining unlabeled samples $K^{\left(\mathrm{Rest}\right)}$ are set to 20, 10, and 0, respectively.

For PanNuke, the initial learning rate for the semi-supervised stage is 0.0002. Every 5 training epochs, the learning rate is decreased to 0.6 of its current value. Batch size is set to 64, with $N_{i}$ set to 1500, $K^{\left(\mathrm{Conf}\right)}$ set to 300, $K^{\left(\mathrm{Hard}\right)}$ set to 300, and $K^{\left(\mathrm{Rest}\right)}$ set to 0.

Each iteration of the mixed-supervised stage comprises 20 training epochs. The weight_decay parameter is set to 8 × 10^−5^, and Adam optimizer [35] is utilized to optimize model parameters via gradient descent.

The specific parameter settings for each dataset can be found in Table 4.

#### 2.7.3. Evaluation Metrics

In the experiment, the overall accuracy (OA) and class-specific accuracy are utilized to evaluate the performance of tumor cell classification. The specific calculation equations are as follows:

(3) $$\mathrm{OA}=\frac{N^{\left(\mathrm{correct}\right)}}{n_{0}^{\left(U\right)}-K^{\left(\mathrm{Hard},\mathrm{all}\right)}}$$

(4) $$OA^{\left(c\right)}=\frac{N^{\left(\mathrm{correct},c\right)}}{n_{0}^{\left(U,c\right)}-K^{\left(\mathrm{Hard},c\right)}}$$

where OA represents the overall classification accuracy, $N^{\left(\mathrm{correct}\right)}$ denotes the number of correctly classified samples among all open data, $n_{0}^{\left(U\right)}$ represents the total number of samples in all open data. $OA^{\left(c\right)}$ indicates the classification accuracy of cells belonging to class c in the open data, $N^{\left(\mathrm{correct},c\right)}$ represents the number of correctly classified cells of class c, and $n_{0}^{\left(U,c\right)}$ represents the total number of cells in class c. $K^{\left(\mathrm{Hard},\mathrm{all}\right)}$ represents the number of samples annotated through human-computer interaction online, and $K^{\left(\mathrm{Hard},c\right)}$ denotes the number of cells of class c annotated through human–computer interaction online. As real-time online annotation can be considered as samples with labels, this part of the samples is not included in the calculation of the metrics.

#### 2.7.4. Environment Configuration

All algorithms are implemented using PyTorch 2.5.1 and run on a machine with 43GB of RAM and RTX 2080Ti GPU.

## 3. Results

### 3.1. Analysis

The performance of the MIX-CC framework is evaluated against baseline algorithms using quantitative metrics across three distinct datasets, as summarized in Table 5, Table 6 and Table 7. The results reveal that MIX-CC consistently surpasses other methods in all three datasets, showing substantial improvements over both fully supervised and semi-supervised learning approaches. This consistent outperformance underscores the effectiveness and robustness of the MIX-CC framework in various classification scenarios.

**Figure 5:**
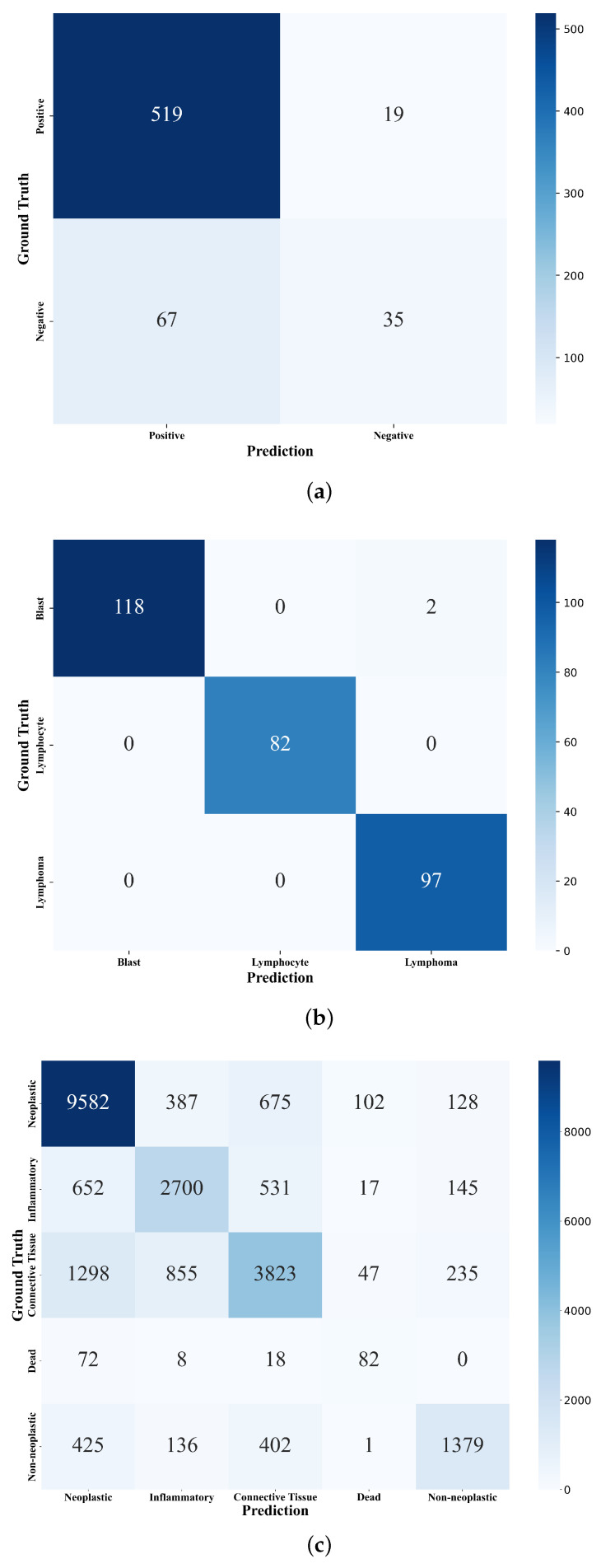
Confusion matrices of MIX-CC for (**a**) LUSC, (**b**) BloodCell, and (**c**) PanNuke datasets.

In LUSC, MIX-CC reaches 86.56% for OA, as indicated in Table 5. The confusion matrix for LUSC (Figure 5a) reveals a noticeable drop in the accuracy of negative cells, while the accuracy of positive tumor cells improves. This could be attributed to the higher difficulty in distinguishing tumor cells, leading the model to misclassify negative tumor cells as positive ones with high confidence. The number of such hard samples recognized by the model is minimal, thus the online annotation fails to sufficiently correct the model.

In BloodCell, MIX-CC achieves a classification accuracy of 99.33%, with the highest accuracy observed for each individual class. Particularly, it achieves perfect performance on lymphocyte and lymphoma cells. The confusion matrix for BloodCell (Figure 5b) highlights the model’s excellent performance in distinguishing all classes, with no significant misclassifications. The superior performance on this dataset can be attributed to the relatively simple background, the small number of cells, and the significant difference between cells and the background, with minimal cell occlusion. The results are shown in Table 6.

In PanNuke, MIX-CC reaches 74.12% for OA, which is nearly 17% higher than all networks in the baseline algorithms. However, the relatively lower performance in this case can be attributed to the larger dataset size, more complex backgrounds, greater cell occlusion, and less distinction between cells and the background. The confusion matrix for PanNuke (Figure 5c) shows that while the model performs well overall, there is a higher rate of misclassification between certain cell types, indicating that the complexity of the dataset impacts the model’s performance. The results are presented in Table 7.

The online annotation mechanism introduced by MIX-CC addresses the issue of unreliable pseudo-labels for “hard” samples. Moreover, the workload of human–computer interactive online annotation is significantly reduced compared to manually annotating all open data by experts. The model can automatically recognize cells and select “hard” samples for human annotation. The results on three datasets demonstrate the effectiveness of the MIX-CC framework.

#### Convergence Analysis

**Figure 6:**
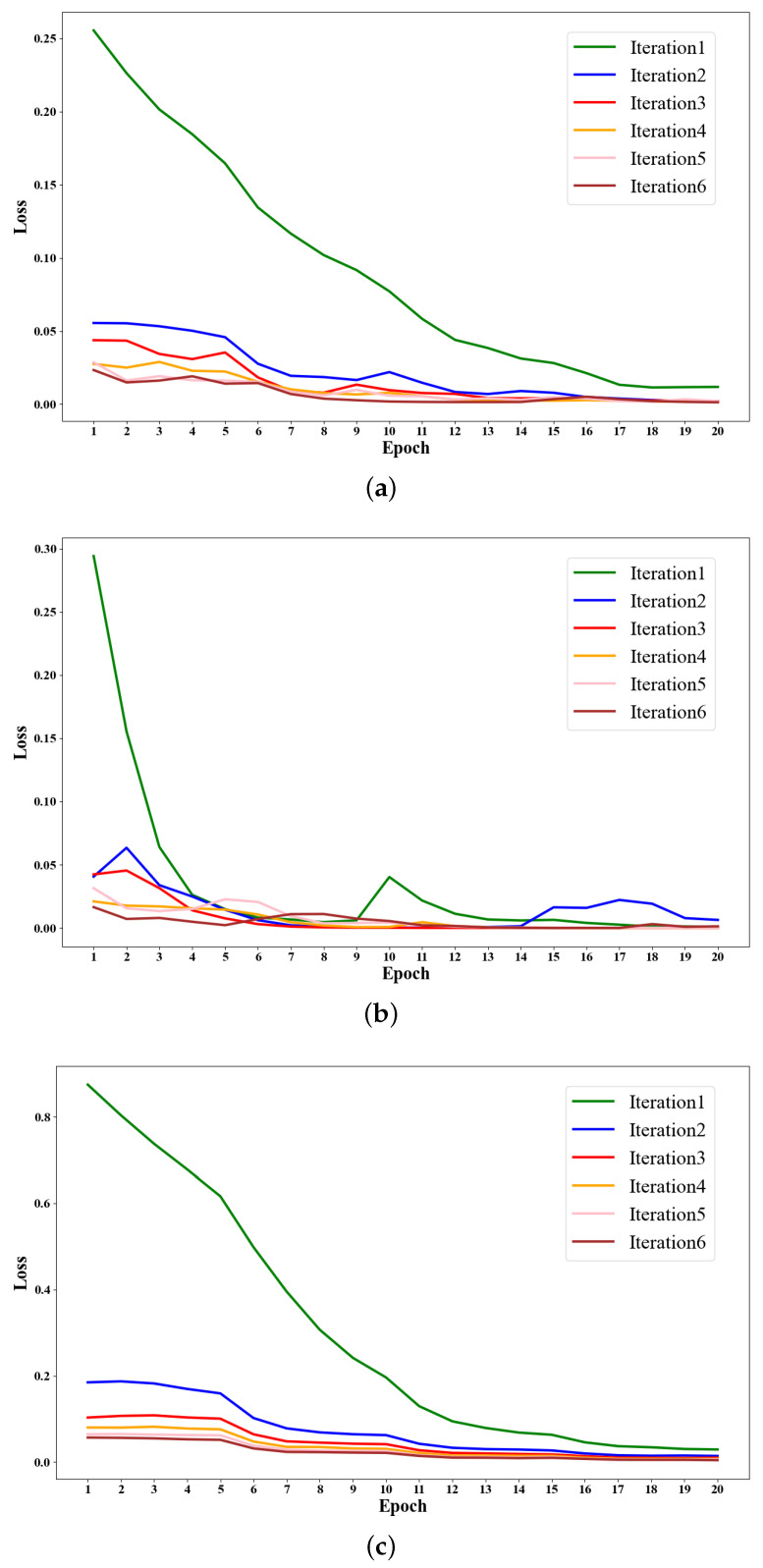
Training convergence curves of MIX-CC for (**a**) LUSC, (**b**) BloodCell, and (**c**) PanNuke datasets in the first six iterations.

Convergence analysis was performed on the MIX-CC model training across various iterations for the three datasets. For illustration, the convergence curves for the first six iterations are presented in Figure 6. From the figure, it can be seen that the MIX-CC loss decreases steadily as the training epochs increase, with this smooth decline observed across all three datasets. This gradual reduction in loss over iterations highlights the model’s stability and convergence.

### 3.2. Ablation Study

To investigate the impact of the “human-in-the-loop” mechanism on cell classification tasks, we conducted ablation experiments on three datasets, referred to as SEMI-CC, as presented in Table 8, Table 9 and Table 10. In this experiment, the “human-in-the-loop” mechanism was deliberately removed, meaning that the selection and processing of “hard” samples, which are those for which the model’s prediction is least confident, was not taken into account. In the absence of this mechanism, only high-confidence unlabeled samples were selected and considered as reliable for inclusion in the training set. These high-confidence samples, along with their corresponding pseudo-labels, were merged with the existing labeled samples, forming an updated training set. This new training set was then used to train the next iteration of the real-time classification model. The omission of the “hard” sample selection process allowed us to examine the performance of the model in a more simplified setup, without the added complexity of integrating challenging, uncertain samples into the learning process.

For LUSC, MIX-CC achieves 86.56% for OA, showing a notable enhancement of 14.69% compared to SEMI-CC. For BloodCell, MIX-CC achieves 99.33% for OA, which represents a 5.02% improvement over SEMI-CC. Similarly, for PanNuke, MIX-CC demonstrated a relative improvement of 15.5% over SEMI-CC, with enhanced classification accuracy across all categories. These results clearly demonstrate the effectiveness of incorporating the “hard” sample selection mechanism, which has significantly enhanced the model’s ability to identify and classify challenging instances. By focusing on these hard samples, the model has become more adept at handling edge cases and has thereby improved its overall classification performance. The inclusion of this mechanism enables the model to not only refine its understanding of rare or complex cases but also to generalize better across a variety of data distributions, making it a more robust and reliable classifier.

## 4. Discussion

The MIX-CC framework demonstrates significant improvements in cell classification accuracy by leveraging both labeled and unlabeled data through semi-supervised learning and integrating human-in-the-loop annotation. This approach yields high overall accuracy across datasets, such as BloodCell (99.33%), LUSC (86.56%), and PanNuke (74.12%), showcasing its effectiveness in diverse histopathology applications.

A key strength of MIX-CC is its improved classification accuracy, achieved by optimizing the model with confident pseudo-labels and expert corrections. The “human-in-the-loop” mechanism ensures that misclassifications are corrected by pathologists, allowing the model to adapt to complex datasets.

Clinically, MIX-CC has great potential for improving diagnostic accuracy and streamlining workflows, reducing the need for manual annotation and allowing pathologists to focus on more complex cases. This mechanism is particularly well-suited for large-scale, open pathology datasets, enabling broader real-world applications. By leveraging this mechanism, the model can effectively handle the challenges posed by vast amounts of unlabeled data, optimizing its predictions iteratively and reducing the need for exhaustive manual annotations. Furthermore, the method enhances classification accuracy by pretraining the backbone using prior human annotations and refining the model through reliable pseudo-labels and online human annotations. This dual-phase process facilitates the precise interpretation of open data, making the approach ideal for large-scale, practical applications in clinical and research settings. By continuously refining the model with real-time annotations, MIX-CC ensures that the classification performance remains robust even in the presence of diverse, complex data from different sources.

Our algorithm has some limitations, such as the restricted feature representation capacity of the backbone. In near future, we plan to explore more advanced deep learning architectures for the backbone to enhance the overall generalizability and adaptability of the algorithm across diverse datasets. Apart from that, we could develop some explainability tools to improve the adoption of our method, MIX-CC, in clinical practice.

## 5. Conclusions

To overcome the limitations of traditional supervised learning methods in the complex task of cell classification, this paper proposes MIX-CC, a mixed-supervised approach that combines semi-supervised learning with “human-in-the-loop” guidance for cell classification in histopathology images. This method introduces an advanced sample selection mechanism which assigns highly confident unlabeled samples for semi-supervised optimization while unreliable samples are sent for online annotation correction.

The experimental results show that our algorithm performs well across different types of pathological stained slides and various cell types. Furthermore, the “human-in-the-loop” combined with semi-supervised learning in the mixed-supervision mechanism outperforms the pure semi-supervised approach. The proposed model is expected to gain abilities if more powerful backbone is applied. In clinical practice, our algorithm can assist pathologists in rapidly interpreting the continuously collected pathological slides.

## Figures and Tables

**Table 2:** Nomenclature.

Symbols	Notations
$H\in N^{*}$	height of the image
$W\in N^{*}$	width of the image
*B*	number of channels in the image, where B=3
${\underline{X}}_{i}\in R^{H\times W\times B}$	the input cell image patch
$n^{\left(L\right)}\in N^{*}$	the number of images in the training set
$n_{0}^{\left(L\right)}\in N^{*}$	the initial number of labeled samples
$n_{0}^{\left(U\right)}\in N^{*}$	the initial number of unlabeled samples
${\underline{X}}_{j}^{\left(L\right)}\in R^{H\times W\times B}$	the $j^{\;\mathrm{th}}$ image in the labeled dataset
${\underline{X}}_{j}^{\left(U\right)}\in R^{H\times W\times B}$	the $j^{\;\mathrm{th}}$ image in the labeled dataset
$y_{j}$	the label of the $j^{\;\mathrm{th}}$ image in the labeled dataset
${\hat{y}}_{j}$	the prediction of the $j^{\;\mathrm{th}}$ image
$K^{\left(\mathrm{Conf}\right)}\in N^{*}$	the number of “credible” unlabeled samples selected
$K^{\left(\mathrm{Hard}\right)}\in N^{*}$	the number of “hard” unlabeled samples selected
$K^{\left(\mathrm{rest}\right)}\in N^{*}$	the remaining unlabeled samples
$N_{i}\in N^{*}$	the number of unlabeled samples randomly selected in the $i^{\;\mathrm{th}}$ iteration
$K_{i}\in N^{*}$	the total number of unlabeled samples selected in the $i^{\;\mathrm{th}}$ iteration
$m^{\left(U\right)}\in N^{*}$	all selected unlabeled credible sample set

**Table 3:** Model and Algorithm Settings for Cell Segmentation and Classification.

Dataset	Cell Segmentation Model	Fully Supervised Learning Model	Semi-Supervised Learning Model
LUSC	UNeXt	ResNet18, SimCLR	ResNet18, SimCLR
BloodCell	UNeXt	ResNet18, SimCLR	ResNet18, SimCLR
PanNuke	HoVer-Net	ResNet50, SimCLR	ResNet50, SimCLR

**Table 4:** Parameter configuration for model training.

DataSet	LUSC	BloodCell	PanNuke
Epoch	80	80	80
Batch Size	64	64	64
Learning Rate	0.0003	0.0002	0.0002
LR Decay Factor/Epoch	0.6/5	0.8/5	0.6/5
Weight Decay	8 × 10^−5^	8 × 10^−5^	8 × 10^−5^
$N_{i}$	300	300	1500
$K^{\left(\mathrm{Conf}\right)}$	20	20	300
$K^{\left(\mathrm{Hard}\right)}$	20	20	300
$K^{\left(\mathrm{Rest}\right)}$	0	0	0

**Table 5:** Overall classification accuracy (OA) on LUSC dataset.

Method	Positive	Negative	Total
SimCLR [33]	70.09%	55.51%	67.38%
ResNet50 [31]	73.38%	53.81%	69.74%
ResNet18 [31]	74.25%	51.69%	70.06%
MIX-CC	96.47%	34.31%	86.56%

**Table 6:** Overall classification accuracy (OA) on BloodCell dataset.

Method	Blast	Lymphocyte	Lymphoma	Total
SimCLR [33]	95.89%	92.75%	88.39%	92.26%
ResNet50 [31]	94.52%	96.38%	86.45%	92.26%
ResNet18 [31]	96.58%	94.20%	90.97%	93.85%
MIX-CC	98.33%	100.00%	100.00%	99.33%

**Table 7:** Overall classification accuracy (OA) on PanNuke dataset.

Method	Neoplastic	Inflammatory	Connective Tissue	Dead	Non-Neoplastic	Total
SimCLR [33]	73.07%	50.96%	46.91%	41.69%	27.28%	55.69%
ResNet50 [31]	72.43%	54.24%	48.30%	46.34%	36.13%	57.57%
ResNet18 [31]	73.60%	51.63%	44.84%	46.78%	33.46%	56.27%
MIX-CC	88.12%	66.75%	61.09%	45.56%	58.86%	74.12%

**Table 8:** Overall classification accuracy (OA) on LUSC dataset.

Method	Positive	Negative	Total
SEMI-CC	78.61%	42.37%	71.87%
MIX-CC	96.47%	34.31%	86.56%

**Table 9:** Overall classification accuracy (OA) on BloodCell dataset.

Method	Blast	Lymphocyte	Lymphoma	Total
SEMI-CC	95.21%	92.65%	92.26%	94.31%
MIX-CC	98.33%	100.00%	100.00%	99.33%

**Table 10:** Overall classification accuracy (OA) on PanNuke dataset.

Method	Neoplastic	Inflammatory	Connective Tissue	Dead	Non-Neoplastic	Total
SEMI-CC	71.43%	51.11%	52.18%	45.23%	43.60%	58.62%
MIX-CC	88.12%	66.75%	61.09%	45.56%	58.86%	74.12%

## Data Availability

Data are unavailable due to privacy restrictions.
